# Association mapping of partitioning loci in barley

**DOI:** 10.1186/1471-2156-9-16

**Published:** 2008-02-18

**Authors:** James Cockram, Jon White, Fiona J Leigh, Vincent J Lea, Elena Chiapparino, David A Laurie, Ian J Mackay, Wayne Powell, Donal M O'Sullivan

**Affiliations:** 1 Centre for Plant Genetics, Breeding and Evaluation, National Institute of Agricultural Botany, Cambridge, UK; 2 CRA – Unità di Ricerca, Sant'Angelo Lodigiano (Lodi), Italy; 3 Crop Genetics, John Innes Centre, Norwich, UK

## Abstract

**Background:**

Association mapping, initially developed in human disease genetics, is now being applied to plant species. The model species *Arabidopsis *provided some of the first examples of association mapping in plants, identifying previously cloned flowering time genes, despite high population sub-structure. More recently, association genetics has been applied to barley, where breeding activity has resulted in a high degree of population sub-structure. A major genotypic division within barley is that between winter- and spring-sown varieties, which differ in their requirement for vernalization to promote subsequent flowering. To date, all attempts to validate association genetics in barley by identifying major flowering time loci that control vernalization requirement (*VRN-H1 *and *VRN-H2*) have failed. Here, we validate the use of association genetics in barley by identifying *VRN-H1 *and *VRN-H2*, despite their prominent role in determining population sub-structure.

**Results:**

By taking barley as a typical inbreeding crop, and seasonal growth habit as a major partitioning phenotype, we develop an association mapping approach which successfully identifies *VRN-H1 *and *VRN-H2*, the underlying loci largely responsible for this agronomic division. We find a combination of Structured Association followed by Genomic Control to correct for population structure and inflation of the test statistic, resolved significant associations only with *VRN-H1 *and the *VRN-H2 *candidate genes, as well as two genes closely linked to *VRN-H1 *(*HvCSFs1 *and *HvPHYC*).

**Conclusion:**

We show that, after employing appropriate statistical methods to correct for population sub-structure, the genome-wide partitioning effect of allelic status at *VRN-H1 *and *VRN-H2 *does not result in the high levels of spurious association expected to occur in highly structured samples. Furthermore, we demonstrate that both *VRN-H1 *and the candidate *VRN-H2 *genes can be identified using association mapping. Discrimination between intragenic *VRN-H1 *markers was achieved, indicating that candidate causative polymorphisms may be discerned and prioritised within a larger set of positive associations. This proof of concept study demonstrates the feasibility of association mapping in barley, even within highly structured populations. A major advantage of this method is that it does not require large numbers of genome-wide markers, and is therefore suitable for fine mapping and candidate gene evaluation, especially in species for which large numbers of genetic markers are either unavailable or too costly.

## Background

Determining the genetic basis of economically important complex traits is a major goal of plant breeding, and has largely been accomplished using linkage mapping of quantitative trait loci (QTL). However, focus is now turning towards the use of association mapping (recently reviewed by [1]), initially applied in human disease genetics. Both approaches rely on the strength of associations between genetic markers and phenotype. However, while linkage analysis searches for associations within populations developed from bi-parental crosses, association mapping utilizes historic patterns of recombination that have occurred within a sample of individuals (e.g. a collection of varieties, landraces or breeders' lines). This has the advantage of allowing existing collections to be screened for many different phenotypes, as well as taking advantage of historical phenotype data from lines thoroughly characterized during variety development. Association mapping is based on the principle that over multiple generations of recombination, correlations only with markers tightly linked to the trait of interest will remain. As a predominantly inbreeding species, cultivated barley is an attractive target for association mapping as its genome contains extensive blocks of chromatin in linkage disequilibrium (LD) [2,3], providing a well-defined haplotype structure from which marker-trait associations can be identified. However, spurious associations between genotype and trait may be detected due to the degree of structure or subdivision within the population, necessitating development of various statistical methods to account for population structure (recently reviewed by [4]).

One of the major divisions within barley germplasm is the distinction between vernalization sensitive and insensitive varieties. Plants with a vernalization requirement require a prolonged period of cold (vernalization) in order to promote subsequent flowering. This is characteristic of the wild ancestors of temperate crops such as barley and wheat. During the spread of agriculture, human selection has resulted in the partitioning of the majority of barley germplasm into spring- and winter-sown varieties which lack or retain a vernalization requirement, respectively. The molecular genetics of vernalization requirement in barley is relatively well characterised (recently reviewed by [5]), and is controlled predominantly by two major loci: *VRN-H1 *and *VRN-H2 *[6]. Spring alleles are thought to be due to deletions spanning putative *cis*-elements in *VRN-H1 *intron I [7-10], or by deletion of part or all of the genomic region carrying the *VRN-H2 *candidate genes [7,11,12]. These well documented genetic determinants of the phenotype make the genetics of vernalization response an attractive test-bed for association mapping in barley.

Spurious associations between genotype and trait due to population sub-structure is widely recognised as a serious obstacle to association mapping. This is likely to be of particular significance in barley, as diversity studies have shown barley germplasm to be highly partitioned, predominantly due to vernalization requirement (spring or winter growth habit) and ear row-number [3,13,14]. Highly stratified populations result in differences in allelic frequency between sub-populations, which may result in false associations between genotype and phenotype in uncorrected regression analysis. Even if association mapping panels are selected within one of these phenotypic classes, sub-structure is still likely to be present due to other factors such as geographic origin and related pedigree. Some of the first association mapping studies in plants were conducted in the model species *Arabidopsis*, and demonstrated the ability to identify previously characterised flowering time loci involved in local adaptation, and hence correlated with population structure [15,16]. As a first step in the application and validation of association genetics to barley, previous studies attempted to identify *VRN-H1 *and *VRN-H2 *[3,17]. However, to date, all such association studies have failed to identify these loci, despite their well characterised genetics and robust phenotypes. Here, we apply a combination of Structured Association (SA) [18] to correct for population sub-structure and Genomic Control (GC) [19] to correct for residual inflation of the test statistic due to unidentified population structure effects and residual confounding arising, for example, from close pedigree relationships among varieties. GC alone is very effective at controlling false positive rates but can result in loss of power in the presence of large effects of population structure [20]. SA will account for population substructure, but is less effective at adjusting for genetic relationships within subpopulations. In addition, identifying the appropriate number of subpopulations for SA analysis can be problematic. We reasoned that the combination of the two methods would negate the deficiencies in each. Although other approaches to association genetics exist, in particular the mixed model approach encoded in the software TASSEL [21] and the principal components method EIGENSTRAT [22], these methods require many more background genetic markers than were available for this study. Using SA + GC, we successfully demonstrate association mapping of *VRN-H1 *and *VRN-H2 *in a collection of European barley germplasm, despite the prominent role of these genes in defining germplasm structure, yet we maintain a false positive frequency close to the expected value elsewhere in the genome.

## Results

### Genome-wide Marker Profiling

In order to aid subsequent population structure and association analysis, a combination of Sequence-Specific Amplification Polymorphism (S-SAP), SSR and gene-based markers were screened across the complete varietal collection. S-SAP techniques generated 129 polymorphic markers, giving a mean S-SAP minor allele frequency (M.A.F.) of 0.22, close to the expected value of 0.25 given a uniform distribution. In addition, three *VRN-H1 *region SSRs were screened (*EBmatc0003, GMS027 *and *Bmag0222*). Four alleles were identified for *EBmatc0003*, with a heterozygosity value of 0.504. *GMS027 *(12 alleles) and *Bmag0222 *(5 alleles) displayed heterozygosity values of 0.62 and 0.64, respectively. The remaining SSRs are distributed five per chromosome, and are described in detail by [14]. One of these, *Bmag0135 *(chromosome 7H), was removed from the final analysis due to the high level of missing data. InDels amplified from *HvCSFs1 *and *HvPHYC *were also scored across the varietal set. Interestingly, 5 % of barley varieties were heterozygous for the *HvPHYC *assay, although genome wide markers did not indicate heterozygosity to be present elsewhere in the genomes of these lines. Previous studies have shown that two *HvPHYC *pseudogenes (*HvPHYC*Ψ*1a *and *HvPHYC*Ψ*1b*) map to distinct genetic loci relative to the functional *HvPHYC *gene, and represent duplications of the 5' end of the gene [23]. However, the assay employed here is based on primers at the 3' end of the gene, suggesting the presence of both InDel forms may be due to detection of an additional duplication event specific to these varieties.

### Extent of LD on Chromosomes 4H and 5H

**Figure 1:**
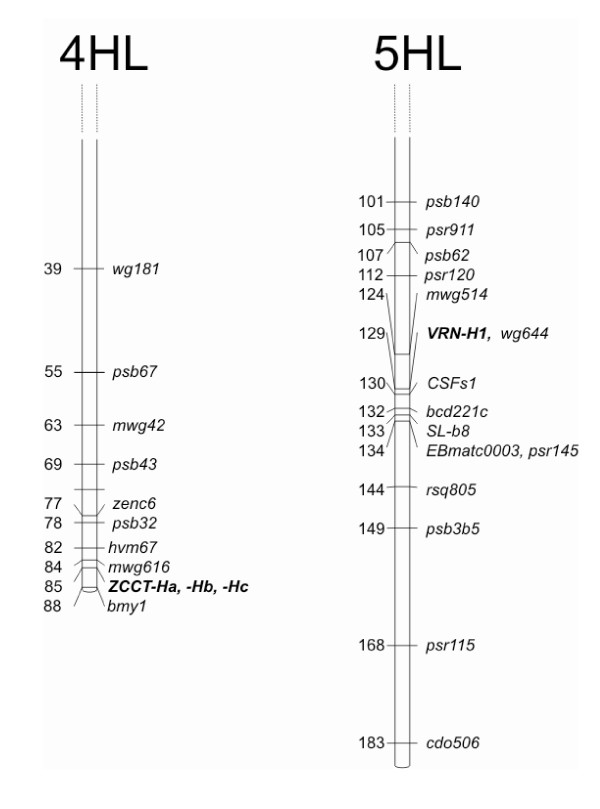
**Genetic mapping in the 'Igri' × 'Triumph' population**. Genetic mapping of *ZCCT-Ha, -Hb, -Hc, HVM67, VRN-H1, HvCSFs1*, and *EBmatc0003 *in the 'Igri' × Triumph' DH population [6].

**Figure 2:**
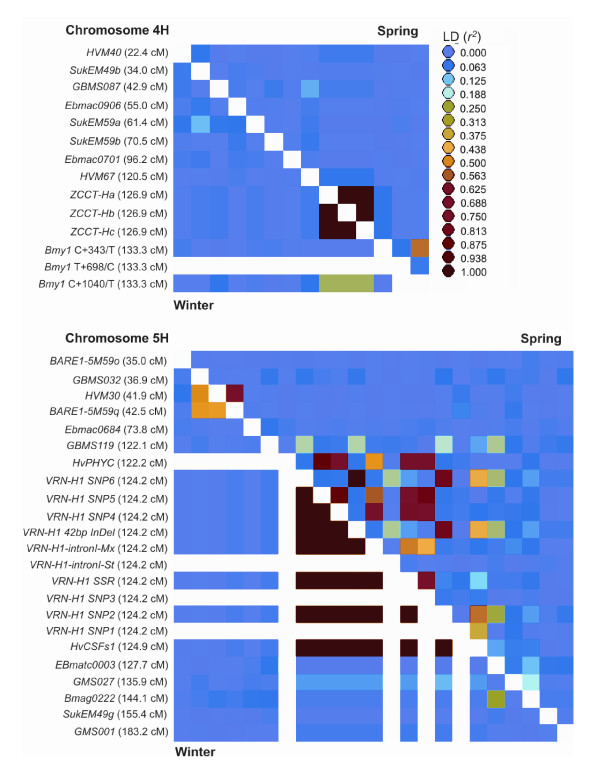
**LD on chromosomes 4H and 5H**. LD matrix of spring (above the diagonal) and winter (below the diagonal) European barley varieties, as measured by *r*^2 ^(colour coded by magnitude). Markers along the x-axis follow the same order as those indicated on the y-axis. Horizontal and vertical white blocks represent uncalculated data, due to invariant genotypes at these loci. Genetic map positions are from the consensus map of Varshney *et al *[24], after integration of additional markers where necessary. The scarcity of anchor markers close to *HvPHYC *in the 'Dicktoo' × 'Morex' genetic map [23] for integration into the consensus map, resulted in incorrect estimations of map position. Therefore, *HvPHYC *has been positioned 2 cM proximal to *VRN-H1*, as described by Szűcs *et al *[23].

Given the haplotype evidence for a local genetic bottleneck caused by a reliance on a single *VRN-H1*/*VRN-H2 *allelic configuration conferring winter GH [9], a selection of markers spanning appropriate regions of chromosomes 4H and 5H were used to assess any constraint in decay of LD between the two germplasm pools. In order to estimate LD decay, markers were integrated into the barley consensus map of [24]. *VRN-H1 *and the candidate *VRN-H2 *genes *ZCCT-Ha, -Hb *and *-Hc *were mapped to chromosomes 5HL and 4HL, respectively (Figure 1), as previously shown [7,11,23,25]. In addition, *HVM67 *mapped 3 cM proximal to the *ZCCT-H *gene cluster, while *HvCSF5s1 *and the SSR marker *EBmatc0003 *both mapped to chromosome 5HL, 1 and 5 cM distal to *VRN-H1*, respectively. Matrices of *r*^2 ^were derived separately for spring and winter varieties using bi-allelic markers only (Figure 2). As expected, the levels of LD on chromosome 4H reveal strong associations between the three closely linked *ZCCT-H *genes (*r*^2 ^= 1.0), in both the spring and winter subpopulations. However, LD between the *ZCCT-H *locus and flanking markers is only found to extend to *Bmy1*, 6.4 cM distal: the *Bmy1 *single nucleotide polymorphism (SNP) T+698/C is invariant in the winter population, while C+1,040/T is in LD with the *ZCCT-H *locus in winter (*r*^2 ^= 0.313) varieties. No other significant levels of LD were identified on chromosome 4H. The LD matrix for chromosome 5H shows very strong levels of LD within *VRN-H1 *(for which ten markers were assayed, see Methods)in winter varieties (*r*^2 ^= 1), with 3 invariant markers (*VRN-H1-SNP1*, *VRN-H1-SNP3 *and *VRN-H1*-*intronI-St*). However, breakdown of LD within *VRN-H1 *can be seen in spring varieties, although high degrees of correlation are still found (eg. *r*^2 ^= 1 between *VRN-H1-SNP6 *and *VRN-H1*-*42bp-InDel*). Interestingly, both *HvPHYC *(2 cM proximal to *VRN-H1*, invariant in winter cultivars) and *HvCSFs1 *(0.7 cM distal to *VRN-H1, r*^2 ^= 1) are in strong LD with *VRN-H1 *markers in the winter sub-population, although this breaks down (particularly in the case of *HvCSFs1*), in the spring population. Evidence of LD is also seen on the short arm of chromosome 5H, in spring and winter cultivars (between *HVM30 *and *BARE1-5M59q*), with *GBMS0032 *also showing LD with both *HVM30 *and *BARE1-5M59q *in winter germplasm.

### Population Structure and Association Mapping

**Figure 3:**
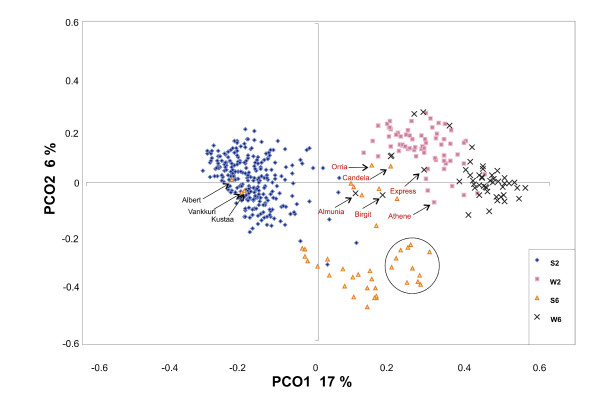
**Principal coordinate analysis of 416 barley varieties**. Growth habit (S = spring, W = winter) and ear-row number (2 = 2-row, 6 = 6-row) are indicated. *VRN-H1 *haplotype designations are previously described [9]. 'Almunia', 'Athene', 'Birgit' and 'Express' (*VRN-H1 *haplotype 5C) are the only four winter varieties that do not belong to haplotype 1A. All included members of the spring *VRN-H1 *haplotype 1B form a sub-cluster within 6-row spring varieties (circled). The proportion of the total variation accounted for by component 1 (PCO1) and 2 (PCO2) is shown in brackets.

To help address false association between markers and phenotype, population substructure within the barley varietal collection was initially investigated using Principal Coordinate Analysis (PCoA). Scatterplots showed distinct clustering according to GH (winter/spring) and row-number (2-row/6-row), identifying four main subgroups: (i) spring 2-row (ii) winter 2-row (iii) spring 6-row (iv) winter 6-row (Figure 3). Some noteworthy outliers can be seen: (i) Three 6-row spring varieties ('Albert', 'Kustaa' and 'Vankkuri') are found within the relatively compact 2-row spring group. However, a search of the ECP/GR European Barley Database [26] returns conflicting row-number data for independent entries for each of the above varieties, suggesting that these varieties may contain passport data errors and require phenotypic verification. (ii) The winter varieties form a notably compact group, with the exception of two outliers ('Almunia' and 'Birgit'). These are found within, but towards the edge of, the spring cluster and represent two of just four winter varieties ('Athene', 'Almunia', 'Birgit' and 'Express') which possess the rare winter *VRN-H1 *haplotype 5C, with the remaining winter varieties belonging to haplotype 1A [9]. The confirmation of winter GH in lines containing *VRN-H1 *haplotype 5C [9] suggests that despite a significant proportion of their genetic background being of spring origin, a winter phenotype has been retained. (iii) Varieties belonging to *VRN-H1 *haplotype 1B form a sub-division within 6-row spring barley, clustering towards the winter cloud and may reflect the close pedigree relationships between members of this haplotype (Additional File 1). Phenotypic analysis of two varieties initially classified as facultative ('Candela' and 'Orria') under long-day photoperiod and non-vernalizing temperatures indicate they possess a spring GH (data not shown). Interestingly, both clustered within the winter group, suggesting they have a predominantly winter genetic background while retaining a spring GH, presumably due to the spring alleles observed at *VRN-H1 *[9].

**Figure 4:**
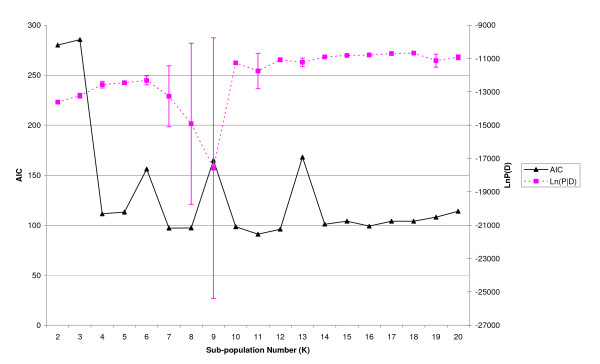
**Identifying the appropriate sub-population number (K)**. Sub-population number (K) against Ln(P|D) ± 1 SD (100,000 burn-in and 900,000 MCMC iterations, 2 replicates) and Akaike's Information Criterion (AIC).

PCoA showed the first and second principal components accounted for only 17 % and 6 % of the genetic variation, respectively, suggesting significant substructure might remain. To help account for this, the Bayesian model-based clustering method implemented by the software STRUCTURE was employed to detect population structure within the varietal collection. The major phenotypic distinctions in cultivated barley suggest K = 4 (corresponding to the four possible GH and row-number combinations) might be enough to capture most of the sub-population stratification present, although the PCoA conducted above suggests that further sub-structure exists. In order to explore population stratification, values for K in the range 1 to 20 were evaluated in terms of Ln(P|D) and AIC (Figure 4). We noted that the value of Ln(P|D) rose steadily up to K = 6, with good duplication of matrices (measured as r) until K = 8. However replication of Ln(P|D) was poor for K in the range 7 to 9, despite multiple replicate analyses. Above K = 9 Ln(P|D) was more stable, but it proved impossible to obtain high correlation between replicate matrices. Thus, analysis of values of Ln(P|D) did not permit clear interpretation. However, AIC showed that variation in winter/spring phenotype was adequately described at K = 4. We reason that since it is spurious association between trait and population structure for which we wish to control then, *for this trait*, the appropriate minimum value of K is 4. Values of K greater than 6 would be difficult to use reliably since they either show poor agreement between replicates in terms of Ln(P|D) and/or in terms of correlation between replicate matrices.

**Figure 5:**
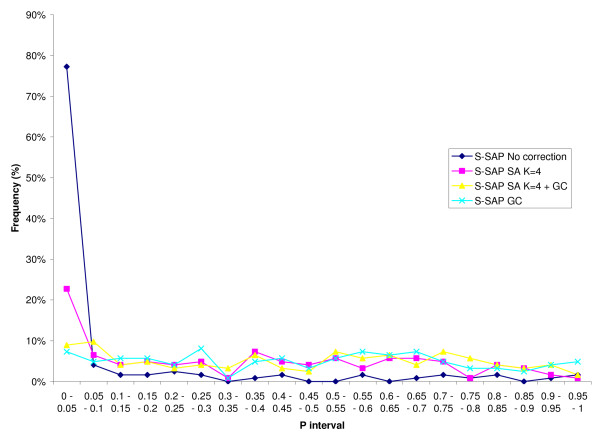
**Frequency distribution of p values for unmapped S-SAP markers**. p values shown without correction, with Structured Association (SA) with 4 sub-populations (K = 4), SA (K = 4) + Genomic Control (GC), and GC alone.

**Figure 6:**
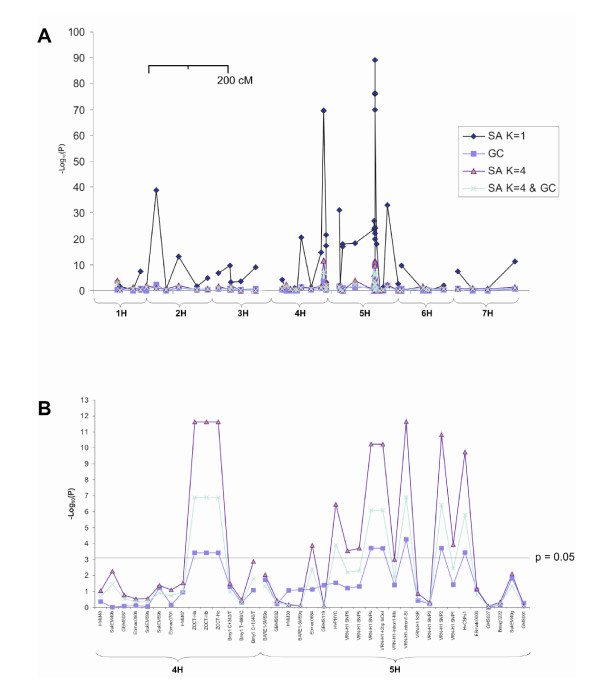
**Association mapping of GH in barley**. Association of GH with mapped genetic markers using association (SA K = 1), Genomic Control (GC), SA with a population sub-structure = 4 (SA K = 4) and SA with population sub-structure = 4 plus Genomic Control (SA K = 4 & GC). GC was performed using the robust mean of LRT for the unmapped S-SAP markers. **(A) **All mapped markers. Distance between markers is proportional to scale (cM). **(B) **Detailed view of mapped markers on chromosomes 4H and 5H (SA K = 1 not shown). Associations between marker and GH are considered significant above p = 0.05/marker number (Bonferroni correction). Distance between markers is not to scale.

We used K = 4 in SA analysis [18,27] with and without GC, with GH designations used as a two-state categorical phenotype. For comparison we repeated the analysis without implementing SA (K = 1). Logistic regression against population membership, and subsequently on marker genotypes (all mapped S-SAP and bi-allelic genome-wide markers), was performed to identify statistically significant marker-trait associations after adjustment for population structure [18,27]. Strength of single marker associations were calculated with and without the inclusion of population structure effects in the model for K equal to 1 and 4. Additional control for the high level of spurious association predicted in highly structured samples was implemented using Genomic Control [19,28], whereby marker chi-squared values (1 d.f.) were divided by the estimate of their robust mean [(median χ^2^)/0.455] of the S-SAPs. Results of these analyses are summarised in Figures 5 and 6. Comparison of statistical corrections applied to the unmapped S-SAP markers shows that without correction, 77 % of the markers show association at p = 0.05, but this falls rapidly with the application of SA (23 %), SA and GC together (9 %) and GC alone (7 %) (Figure 5). Figure 6 shows the effect over mapped markers on chromosomes 4H and 5H. The critical observation is that SA appears to help distinguish between association which is dependent or independent of sub-population membership. Figure 6B shows significant associations were observed on chromosomes 5H and 4H (on which *VRN-H1 *and *VRN-H2 *are located, respectively). Amongst the mapped markers, fourteen associations with GH were significant at p < 0.05 (Bonferroni adjusted) after adjusting for population structure alone. Subsequent implementation of GC reduced this to nine markers: four within *VRN-H1 *(*VRN-H1-intronI-St*, *VRN-H1-SNP2*, *VRN-H1-SNP4*, *VRN-H1-42bp-InDel*), the *VRN-H2 *candidate genes *ZCCT-H*a, *-Hb, -Hc*, as well as *HvCSFs1 *(0.7 cM distal to *VRN-H1*) and *HvPHYC*. No additional markers were found to be significant.

## Discussion

For association mapping to be possible, LD must be present in the collection of individuals under study, with the levels of LD varying according to species and locus investigated. Previous studies in maize and the predominantly self-pollinating model species *Arabidopsis *and rice have demonstrated strong levels of LD surrounding genes controlling flowering time and disease resistance, extending from tens of kb up to 1 cM [14,23,25,27,29-32]. In the temperate crop barley, LD has been identified over a ~200 kb region encompassing the *Ha *locus controlling grain hardness [33], and up to a distance of 5.5 cM from a gene conferring resistance to the barley yellow mosaic virus (BMYV) complex [34]. Here we estimate LD to extend at least 0.7 cM from *VRN-H1 *and 6.4 cM from *VRN-H2*. This is within a similar range of LD previously identified surrounding selected traits in barley, and helps to indicate the scale of marker saturation required to permit future association mapping of loci of interest in barley. High levels of LD were observed within *VRN-H1 *over distances of up to 16.7 kb (winter *VRN-H1 *haplotype 1A), suggesting LD decay within *VRN-H1 *may differ between European germplasm, compared to the low levels suggested to occur in North American germplasm [17].

As an initial inspection of population substructure, PCoA suggested primary division within the sample investigated is due to GH. Division within GH pools due to row-number was more distinct within the spring germplasm. Furthermore, notable sub-clustering was observed for varieties belonging to the spring *VRN-H1 *haplotype 1B [9], found predominantly in Scandinavian varieties. PCoA clearly showed that sub-structure existed within the varietal population and would therefore need to be adequately accounted for during association analysis. It is generally acknowledged that establishing a value for K (the number of sub-populations) prior to association analysis, is not a trivial exercise [35,36]. To identify the appropriate value of K for use in this study, we attempted to find a maximum for Ln(P|D) (a measure of the maximum likelihood of the specific sub-population model as a description of these data) and also to minimise AIC (which finds the value of K at which the phenotypic variation is best accounted for by the sub-population membership matrix). Neither approach gave an unambiguous answer but instability in determining the Q matrix suggested K > 6 was unsafe, while AIC suggested most of the phenotypic variation was described at K = 4. Varietal membership to the four groups does not correspond to the expected winter/spring, 6-row/2-row combinations, although 96 % of sub-population 1 are 2-row spring, and sub-population 3 contains 87 % of all winter varieties (data not shown). SA with K = 4 appears to have eliminated a high proportion of false positive results: out of 61 markers tested 40 were significant in uncorrected data at p = 0.05 (Bonferroni corrected), falling to 14 after SA was applied (Figure 6). However, in many situations, prior knowledge as we have described above will not be available. In these circumstances we believe that exploration of Ln(P|D) and AIC values could give useful guidance.

Association mapping by logistic regression without correction for structure or for inflation of λ due to multiple testing showed significant association across chromosomes 4H and 5H, while 77 % of marker/trait associations using the unmapped S-SAP markers had an observed p value ≤ 0.05 (Figure 5 and 6A). Correction using structured association alone (K = 4) resulted in a large reduction in associated markers, presumably due to a selective reduction of spurious associations, reflected in the reduction from 77 % to 23 % of markers showing association with p ≤ 0.05 (Figures 5 and 6B). The addition of genomic control to SA analysis resulted in the elimination of an additional five markers on chromosomes 4H and 5H; applied to the genome wide marker set, the proportion of markers with p ≤ 0.05 was reduced to 9 %. Interestingly, GC alone reduces the number of markers significant at p ≤ 0.05 slightly more (7 %) than when used in conjunction with SA (9 %). We attribute this to the discriminating nature of the SA correction: GC alone (based on a robust mean of λ = 24.7) reduces the test statistic for all markers by an equal proportion; in contrast SA selectively reduces λ at each marker using a correction specifically tailored to the sub-population fractional membership of each individual in the experimental population. SA reduced background association such that the robust mean of λ falls to 1.8. Subsequent GC correction is therefore much less stringent because the general inflation of the test statistic has already been largely removed by SA. In summary, we believe the approach undertaken gives a higher objective threshold for significance and therefore reduces the number of potentially associated markers for consideration. Despite GH representing one of the major divisions within barley germplasm, association mapping using the combined SA and GC approach identified markers within *VRN-H1 *and candidate *VRN-H2 *genes as significantly associated with GH after applying statistical correction for the population structure they largely define. Of all the *VRN-H1 *markers investigated here, the *VRN-H1-intronI-St *assay (which tests for the absence of large intron I deletions within a 'vernalization critical' region associated with the recessive winter *vrn-H1 *allele) was previously found to show strong correlation with GH [7-9]. SA found this assay to show the highest association with GH (Figure 6B), demonstrating the statistical approaches undertaken here agree with previous studies aimed at determining functional polymorphism at *VRN-H1*. Five additional *VRN-H1 *markers showed highly significant (but lower) p values, although since they are not within the 'vernalization critical' region of *VRN-H1*, their significance is most likely due to the strong LD identified within the gene.

The identification of markers strongly associated with GH, 0.7 cM distal (*HvCSFs1*) and 2 cM proximal (*HvPHYC*) [23] to *VRN-H1 *illustrates this locus could potentially have been identified by a genome-wide scan even in the absence of the candidate genes assayed here, given a density of approximately one marker every 1 cM. This level of marker saturation is yet to be routinely achieved in large genome crops, although coverage approaching this density appears feasible in barley [3]. Such practical limitations for the use of association genetics in plants suggests it is better suited to fine-mapping, after localization of the trait of interest by QTL mapping [1]. This approach would have been applicable here, even with no prior knowledge of *VRN-H1 *and candidate *VRN-H2 *genes. Indeed, the association analysis carried out in this study provided a resolution capable of differentiating between intra-genic *VRN-H1 *markers. Previous association mapping studies have failed to identify *VRN-H1*, despite an average marker density of ~84 SNPs per chromosome [3]; instead, a marker 27 cM proximal to *VRN-H1 *was highly associated with growth habit. Furthermore, the use of a G/C SNP in the 3' UTR of *VRN-H1 *in an association study based on a collection of 102 North American barley varieties analysed in conjunction with ~1,100 genome-wide SNPs, showed no correlation with growth habit [17]. The failure to detect *VRN-H1 *and *VRN-H2 *in previous association mapping studies may have been due to factors such as sample size, partitioning according to population structure and phenotypic errors. However, even if closely linked markers are available, the power to detect association also depends on marker and trait allele frequencies [37,38]. For example, if the minor allele frequency is very low, or present in a subset of lines not associated with a QTL, the marker is less likely to detect association. This is evident here, where despite the high associations between the majority of *VRN-H1 *markers and growth habit, three failed to detect significant association.

A variety of approaches have been successfully used to detect marker-trait associations in cereals. Thornsberry *et al *[27] pioneered the use of Bayesian modelling of population structure (using STRUCTURE) in conjunction with logistic regression in crop plants in an association study of candidate gene polymorphisms with flowering time variation in maize. In addition to logistic regression, the Buckler group have implemented the General Linear Model (GLM) and Mixed Linear Model (MLM) approaches using TASSEL [39]. GLM has been used by Ravel *et al *[40]; MLM, which incorporates a model of kinship between varieties with complex patterns of shared ancestry, is suitable for applications where larger numbers (some hundreds) of markers are available [41]. The MLM approach with SA was used recently to look for associations with seasonal growth habit in barley [3]. Breseghello *et al *[42] used STRUCTURE to detect population structure and MLM to detect marker trait associations in hexaploid wheat. Kraakman *et al *[43], looking for association with yield and yield stability in spring barley, found no population structure using a Bayesian modelling approach, and instead calculated Pearson correlation coefficients and applied a False Discovery Rate to correct for multiple testing. Although the use of MLM was not possible here (due to the limited marker numbers available), the basic logistic regression with SA reported here is analogous to the logistic regression analysis module available in TASSEL. Our implementation of genomic control follows standards set in human studies [19]. It is, to our best knowledge, the first example of its use to control for residual confounding after adjustment by other means. A similar approach is suggested by Pritchard *et al *[18] in their description of SA, and the use of GC in this manner was made explicit by Price *et al *[22] in a supplementary note to their description of a principal components analysis to correct for stratification.

## Conclusion

The following lessons can be drawn from this proof of concept of association mapping in barley: firstly, even with limited marker sets using the right correction models, it is possible to obtain robust associations between genes and major adaptive traits such as vernalization requirement, that themselves define the boundaries of population strata restraining gene flow. Performing association mapping in a population with severe substructure due to the presence of spring and winter varieties could be avoided by sampling within one GH class. However, sub-structure due to additional factors such as row-number, geographic origin and related pedigrees means population sub-structure is hard to avoid, even when populations are carefully selected. Thus, demonstrating the ability to successfully account for population structure is likely to be important in almost all instances. Secondly, we show that strong LD can be detected between 0.7 and 6 cM from the partitioning loci investigated. Thirdly, discrimination between causative intra-genic markers and those in strong LD versus those presumably more ancient interspersed polymorphisms not in LD with the causative deletions was possible, highlighting the power of association mapping in dissecting a locus of interest. Finally, we demonstrate a novel method by which taking population structure into account, and then correcting using genomic control, association analysis is possible using marker numbers too low to be used in alternative approaches. The validation of this model presented here could be of particular significance to secondary crops, for preliminary association mapping screens, or any other instance where marker number may be limited.

## Methods

### Germplasm, DNA Extraction, Phenotyping and Nomenclature

Four-hundred and twenty-nine spring and winter barley varieties were sampled from thirteen EU countries, collected as part of the GEDIFLUX project to determine the impact of modern breeding on molecular crop diversity [14,44]. DNA extraction protocols, sourcing and phenotyping of growth habit (GH) and further details of plant varieties are previously described [9,45]. A list of all the lines used as well as the *VRN-H1 *and *VRN-H2 *genotypes, recorded growth habit, country of origin and germplasm source, is previously published [9]. Cereal vernalization locus nomenclature follows that described by [46]; the three closely related members of the *ZCCT-H *gene family (*ZCCT-Ha*, *-Hb *and *-Hc*) follow the nomenclature established by [11].

### Genome-Wide Marker Profiling and Candidate Gene Genotyping

The retrotransposon-based marker system, S-SAP, was applied to the complete varietal set using six of the primer combinations described by Leigh *et al *[47] (Sukkula-E0228+Mse-CTA, Sukkula-E0228+Mse-CAG, Sukkula-9900+Mse-CAT, Bare5980+Mse-CTA, Nkita-E2647+MseCTT and Bagy2C0589+MseCAC). The resulting 129 polymorphic markers were used for subsequent population structure and association analysis. Twelve S-SAP markers (1H: BARE1-5M59n, SukEM59c, SukEM59c; 2H: SukEM49k, SukEM59d, NikM62e, BARE1-5M59; 4H: SukEM49b, SukEM59a, SukEM59b; 5H: BARE1-5M59o, BARE1-5M59q, SukEM49g) have previously been mapped [48]. Genotyping of the ten genetic markers distributed throughout the *VRN-H1 *gene(*VRN-H1-SNP1 *to -*SNP6*, *VRN-H1 SSR, VRN-H1-intronI-St, VRN-H1-intronI-Mx *and *VRN-H1-42bp-InDel*) on chromosome 5HL is previously described [9]. Previously described PCR assays [8] to detect the 5.2 kb intron I deletion characteristic of the spring variety 'Morex' (primer pair Intr1/H/F1 and Intr1/H/R1), and the absence of intron I deletions characteristic of the winter variety 'Strider' (primer pair Intr1/H/F3 and Intr1/H/R3) are termed here *VRN-H1*-*intron1-Mx *and *VRN-H1*-*intron1-St*, respectively. InDels within intron XII of *CLEAVAGE STIMULTAION FACTOR subunit 1 *(*HvCSFs1*, GenBank accession 635P2.2) and exon IV of *PHYTOCHROME C *(*HvPHYC*, DQ238106) were genotyped by agarose gel separation of PCR amplicons generated using the following primer pairs: *HvCSFs1-*F1 5'-GACTTGTGAAGCAATATCCAGG-3'/*HvCSFs1*-R1 5'-AGTAAGGGCGTCCCAGACG-3' and *HvPHYC-*F11 5'-AGTTGTCCACCCAGCGCCAG-3'/*HvPHYC-*R4 5'-TCAGAAGTTGCTCTTGCTCGT-3'. The presence/absence of the three *VRN-H2 *candidate genes *ZCCT-Ha*, *-Hb *and -*Hc*, along with an internal amplification control (*HvSNF2*), were assayed as described by Karsai *et al *[12]. Previously published genotype data obtained using the GEDIFLUX barley varietal set for three polymorphic SNPs in the β-amylase gene, *Bmy1*, closely linked to the *VRN-H2 *locus on chromosome 4HL, as well as 35 SSR markers distributed over all seven chromosomes, were also included in the analysis [14,45]. Four additional 5HL SSRs distal to *VRN-H1 *(*Ebmatc0003*, *GMS027 *and *Bmag0222*) were genotyped over the full varietal collection by detection of PCR products tagged by 5' 6-FAM fluorescent labelling of forward primers. PCR products were separated and sized using the AB3100 sequencing platform (Applied Biosystems), according to the manufacturer's instructions. SSR data was processed using GeneMapper v. 3.7 (Applied Biosystems).

### Genetic Mapping

The *VRN-H1 *SNP, T+14,567/C was mapped using the ABI PRISM^® ^SNaPshot^® ^Multiplex Kit (Applied Biosystems) using primers described by [9]. Gene-specific products were amplified from a doubled haploid (DH) population derived from an 'Igri' (winter) × 'Triumph' (spring) cross [6]. *HvCSFs1 *was mapped in the same population using the amplicon size polymorphism described above, while the SSRs *EBmatc0003 *and *HVM67 *were mapped following protocols described by [49] to allow integration of *VRN-H1 *and *VRN-H2 *into a barley consensus map [24]. Genotype data for the S-SAP [49] and GBMS SSR [50] markers previously mapped in 'Steptoe' × 'Morex' were downloaded from the GrainGenes 2.0 database [51] and combined using JoinMap 3.0 [52]. The map position for *HvPHYC *is as previously published [23], 2 cM proximal to *VRN-H1 *in a 'Dicktoo' × 'Morex' cross. Where necessary, genetic markers were positioned within the consensus map [24], following the protocols they describe.

### Estimation of Population Structure

PCoA was carried out as implemented in GenStat v.8 (VSN International, Herts, UK) on a similarity matrix created from all genotype data using a simple matching coefficient. To account for genetic sub-structure in the varietal collection, individuals were allocated to populations, and ancestry coefficients estimated, using the program, STRUCTURE version 2.2 [35,53,54]. Genotypes were presented in haploid format. Results were summarised in matrices of fractional sub-population membership (Q matrices), modeled with a burnin of 100,000 and a run length of 900,000. SSR genotype data was used to define Q matrices, allowing the unmapped S-SAP data to be used as an independent set of markers for genomic control. Agreement between duplicates was assessed in terms of difference in Ln(P|D) between replicates and by calculating the average maximum correlation between all combinations of sub-populations in the two matrices. Where average maximum correlation (r) was less than 0.99, further replicates with burnin of 200,000 and run length of 1,250,000 were performed to attempt to achieve good duplication. All models were fitted without prior population information provided to the algorithm. Two approaches were taken to estimate the appropriate value for K: (i) from the maximum Ln(P|D), as described by [35]. (ii) Using logistic regression to predict the winter/spring phenotype from the Q matrix obtained at each value of K, and finding the point where change in value of Akaike's Information Criterion (AIC) [55] between subsequent values of K was minimised. Logistic regression was performed using R statistical software v2.5.0 [56]. When a final value of K to be fully tested in association analyses had been determined, new Q matrices were calculated using a burnin of 200,000 and run length of 1,250,000.

### Association Testing

SA analyses [18,27] were carried out by logistic regression of GH on estimated coefficients of ancestry (taken from STRUCTURE analysis and on all mapped markers) using R v.2.5.0. For comparison, logistic regression was also carried out directly on markers without first correcting for population structure. Genomic control [19,28] using the 123 unmapped S-SAP markers was used to account for residual confounding remaining after the application of structured association. Strength of single marker associations were measured as -2 ln(λ) (where λ is the likelihood ratio comparing presence and absence of a marker in the analysis) which is asymptotically distributed as a chi-squared distribution with 1 d.f. under the null hypothesis of no association. Significant associations were recorded above the nominal 5 % significance level divided by the number of markers employed, following the standard Bonferroni procedure.

## Authors' contributions

JC performed genotyping experiments, analysed and interpreted data, JC and JW wrote the manuscript; IJM carried out PCoA and devised the association testing strategy, JW and IM performed the LD, population structure and association analyses. FL performed S-SAP genotyping, EC contributed genotype and mapping data; DAL, VJL, IJM, WP contributed ideas, supervisory input and commented on the manuscript. DOS devised the study, co-ordinated the research and co-wrote the manuscript.

## Supplementary Material

Additional File 1 Pedigree relationships between varieties belonging to *VRN-H1 *haplotype 1B. Dashed lines indicate crosses involving more than two parental lines. Cultivars displaying *VRN-H1 *haplotype 1B [9] are highlighted in red and underlined; varieties not included in this study are highlighted in italicised grey. Country and decade of release are indicated, where known. Click here for file
