# Supplementary figures and images for: Association mapping of partitioning loci in barley
Source: BMC Genet. 2008 Feb 18;9:16. doi: 10.1186/1471-2156-9-16 (PMC2276512; doi:10.1186/1471-2156-9-16)

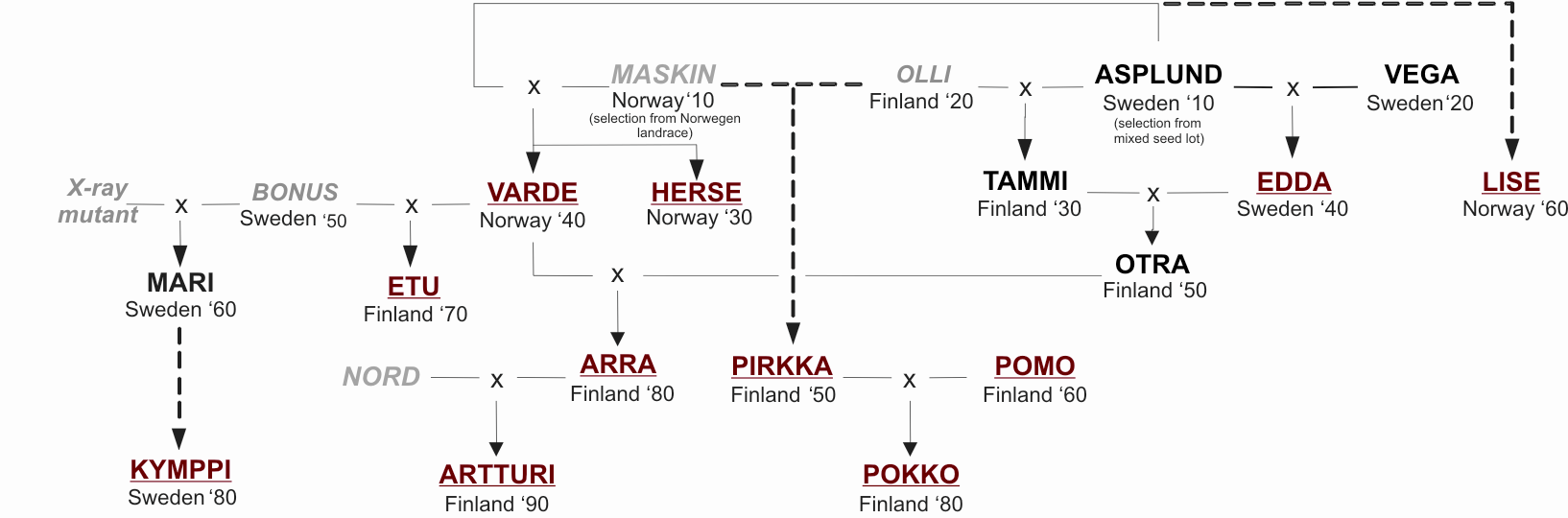

Supplement: Additional File 1 — Pedigree relationships between varieties belonging to VRN-H1 haplotype 1B. Dashed lines indicate crosses involving more than two parental lines. Cultivars displaying VRN-H1 haplotype 1B [9] are highlighted in red and underlined; varieties not included in this study are highlighted in italicised grey. Country and decade of release are indicated, where known. [file 1471-2156-9-16-S1.doc]
